# Simultaneous Editing of Two Copies of *Gh14-3-3d* Confers Enhanced Transgene-Clean Plant Defense Against *Verticillium dahliae* in Allotetraploid Upland Cotton

**DOI:** 10.3389/fpls.2018.00842

**Published:** 2018-06-28

**Authors:** Zhennan Zhang, Xiaoyang Ge, Xiaoli Luo, Peng Wang, Qiang Fan, Guang Hu, Juanli Xiao, Fuguang Li, Jiahe Wu

**Affiliations:** ^1^State Key Laboratory of Plant Genomics, Institute of Microbiology, Chinese Academy of Sciences, Beijing, China; ^2^State Key Laboratory of Cotton Biology, Institute of Cotton Research, Chinese Academy of Agricultural Sciences, Anyang, China; ^3^Institute of Cotton Research, Shanxi Academy of Agricultural Sciences, Yuncheng, China

**Keywords:** *Gossypium hirsutum* L., allotetraploid cotton, simultaneous editing, transgene-clean, CRISPR/Cas9, *Verticillium dahliae*

## Abstract

*Gossypium hirsutum* is an allotetraploid species, meaning that mutants that are difficult to be generated by classical approaches due to gene redundancy. The CRISPR/Cas9 genome editing system is a robust and highly efficient tool for generating target gene mutants, by which the genes of interest may be functionally dissected and applied through genotype-to-phenotype approaches. In this study, the CRISPR/Cas9 genome editing system was developed in *G. hirsutum* through editing the *Gh14-3-3d* gene. In T0 transgenic plants, lots of insertions and deletions (indels) in *Gh14-3-3d* at the expected target site were detected in the allotetraploid cotton At or Dt subgenomes. The results of the PCR, T7EI digestion and sequencing analyses showed that the indels in *Gh14-3-3d* gene can be stably transmitted to the next generation. Additionally, the indels in the At and Dt subgenomes were segregated in the T1 transgenic plants following Mendelian law, independing on the T-DNA segregation. Two homozygous *Gh14-3-3d*-edited plants free of T-DNA were chosen by PCR and sequencing assays in the T1 plants, which were called transgene-clean editing plants and were designated *ce1* and *ce2* in the T2 lines showed higher resistance to *Verticillium dahliae* infestation compared to the wild-type plants. Thus, the two transgene-clean edited lines can be used as a germplasm to breed disease-resistant cotton cultivars, possibly avoiding complex and expensive safety assessments of the transgenic plants.

## Introduction

Cotton is an important economical crop due to its fiber and derivative production, playing crucial roles in human daily life and economical production worldwide. Cotton is planted in approximately 150 countries and is involved in the income of almost 100 million families (Guan et al., 2014; Li and Zhang, 2016). Commercial species of cotton plants are *Gossypium hirsutum* (>90% of world production), *G. barbadense* (3–4%), *G. arboreum*, and *G. herbaceum* (together, 2%). *G. herbaceum* and *G. arboretum* are diploid species with A or D genomes, respectively, while *G. hirsutum* and *G. barbadense* are allotetraploid species, consisting of two sets of subgenomes: At and Dt (Wendel, 1989; Guan et al., 2014). This polyploidization confers many excellent properties to tetraploid cotton, including fiber quality and defense (Li et al., 2015; Zhang et al., 2015). However, the complex genome of allotetraploid cotton brings many challenges for functional analyses and genetic manipulation of cotton genes, mainly due to gene functional redundancy, gene dose effect, and less phenotype of the inserting mutant (Wang Y. et al., 2014; Braatz et al., 2017). Regulation strategies of the expression level, including conventional RNAi and gene overexpression, have been used in the identification of cotton genes, for example, cotton fiber development (Walford et al., 2011; Deng et al., 2012; Tan et al., 2013; Tang et al., 2014; Wang L. et al., 2014; Li Y. et al., 2016; Wan et al., 2016) and stress responses (Min et al., 2013; Li C. et al., 2014; Li Y.B. et al., 2016; Guo et al., 2016). However, “genotype-to-phenotype" approaches are more important in identifying interesting genes. Additionally, cotton genome sequences have been published in many databases, including diploid and allotetraploid species, within recent years (Paterson et al., 2012; Wang K. et al., 2012; Li F. et al., 2014; Yuan et al., 2015; Zhang et al., 2015). Thus, novel gene or genome manipulation urgently needs to be developed to meet the demand for the rapid and precise dissection of cotton gene function.

Recently, the CRISPR (clustered regularly interspaced short palindromic repeat)/Cas9 (CRISPR-associated) genome editing system was developed and has become a robust and highly effective tool for acquiring novel desired mutations in animal and plant. The RuvC-like and HNH domains of the Cas9 protein can form complexes with a synthetic sgRNA, recognizing target sequences that generate double-strand breaks (DSBs) at expected target sites (Jinek et al., 2012; Cong et al., 2013). Those breaks are quickly mended by the innate repair system via non-homologous end joining (NHEJ) and homology-directed repair (HDR). However, the NHEJ repair mechanism frequently creates small insertions and deletions (indels) at the DNA break sites. These indels can generate a frameshift mutation or disrupt important functional domains, damaging or changing the functions of the target genes (Jinek et al., 2012; Cong et al., 2013; Shan et al., 2013). Although the CRISPR/Cas9-mediated genome editing system is a new tool for gene-targeted mutagenesis, it has been successfully applied in genome editing in many plants, such as *Arabidopsis* (Feng et al., 2013, 2014; Fauser et al., 2014), wheat (Upadhyay et al., 2013; Wang Y. et al., 2014), tomato (Brooks et al., 2014), rice (Shan et al., 2013; Wang et al., 2016; Lu et al., 2017; Meng et al., 2017), sorghum (Jiang et al., 2013), maize (Liang et al., 2014), and oilseed rape (Lawrenson et al., 2015; Braatz et al., 2017). In addition, the Cas9 protein, when directed against multiple target sites, can induce mutations simultaneously in different (homologous) sequences, as has already been demonstrated in the tetraploid potato (Wang et al., 2015; Andersson et al., 2017), hexaploid wheat (Upadhyay et al., 2013; Wang Y. et al., 2014), and oilseed rape (Braatz et al., 2017). Very recently, application of genome editing in many important genes, excluding exogenous or endogenous marker genes, has been increasingly presented, such as simultaneously targeted mutagenesis of three homeologs of *TaEDR1* to enhance powdery mildew resistance in wheat (Zhang et al., 2017), editing targeted mutagenesis of *GmFt2a* to delay the flowering time in soybeans (Cai et al., 2017), targeted mutagenesis of γ-aminobutyric acid synthesis genes to increase its levels in *Solanum lycopersicum* (Li R. et al., 2017), and targeting the mutagenesis of two *BnALC* homeologs to reduce seed shattering in oilseed (Braatz et al., 2017), etc. In 2017, there were several papers that documented the development of CRISPR/Cas9-mediated genome editing systems in *G. hirsutum*, primarily through endogenous and exogenous marker genes, including *GhCLA1, DsRed2*, and *GFP*, as well as the *GhMYB25*-like gene (Chen et al., 2017; Janga et al., 2017; Wang P. et al., 2017). Thus, interesting and ecological genes, especially negative regulation genes that function in defense and development, remain to be edited for improving cotton cultivars by the CRISPR/Cas9 genome editing system.

Cotton verticillium wilt, called “cotton cancer,” is a destructive disease, annually leading to 250–310 million US dollars in economic losses in China (Wang et al., 2016). The breed of disease-resistant cultivars is the best measure to prevent plants from pathogen damage by *Verticillium dahliae*. However, few resistant genes or germplasm resources against *V. dahliae* are naturally found in *G. hirsutum*. Thus, it is pivotal to generate novel defense genes or defense mutants using CRISPR/Cas9 targeting the negative regulator of disease-resistance, including cotton *14-3-3c/d, NINJA*, and *CYP82D*, which had high sensitivity against *V. dahliae* infestation as confirmed by RNAi approaches(Gao et al., 2013; Sun et al., 2014; Wang L. et al., 2017). Among these negative defense proteins, the 14-3-3 proteins, a family of conserved regulatory molecules, are found in all eukaryotic cells, which bind to functionally diverse signaling proteins, including kinases, phosphatases, and transmembrane receptors (Obsil et al., 2001). In plants, the 14-3-3c/d proteins have been demonstrated to be negative regulators of BR signaling by regulating two key transcription factors, Brassinazole resistant 1 (BZR1) and Brassinosteroid insensitive 2 (Gampala et al., 2007), and regulating plant response to biotic stress (Nakashita et al., 2003; Vriet et al., 2012; Wang H. et al., 2012). Recently, four 14-3-3 proteins involved in BR signaling were identified in proteomic analysis to have decreased in abundance in cotton plants inoculated with *V. dahliae* (Gao et al., 2013). Silencing of *14-3-3c* and *14-3-3d* through the virus-induced gene silencing (VIGS) method significantly enhanced the resistance of cotton plants to *V. dahliae* (Gao et al., 2013). Thus, the cotton 14-3-3c/d genes can be used as candidate target genes for generating disease-resistant mutants by the CRISPR/Cas9 genome editing system.

In this study, we developed a CRISPR/Cas9-mediated genome editing system in plants with easy and convenient target sequences, by which the cotton target gene, *Gh14-3-3d*, were edited for generating indels at expected target sites. Lots of nucleotide insertions and deletions at the expected sites of the *Gh14-3-3d* target genes were generated in T0 plants induced by the CRISPR/Cas9 genome editing system. We screened 16 T1 lines and acquired two transgene-clean editing plants with homozygous indels in the tetraploid cotton At and Dt subgenomes, designated *ce1* and *ce2* lines in T2 had high resistance to *V. dahliae* compared to the wild type, which can be directly used as a germplasm to breed resistant cultivars and can be avoided to perform safety assessment of transgenic plants, a time-consuming, expensive and tedious process. This successful target gene editing will promote more studies in exploring gene functions of interest and improve agricultural traits.

## Materials and Methods

### Plant Materials, Growth Conditions, and Genetic Transformation

Cotton cultivar CCR35 was used as the transformation receptor for *Gh14-3-3d* gene editing in this study. The hypocotyl of cotton seedlings were cultured in the dark from sterilized seeds in a chamber for 6 days at 30°C and were used as explants for *Agrobacterium*-mediated transformation (Wu et al., 2005). The regenerated plants with perfect roots were directly transplanted in pots with 1:3 vermiculite and organic matter soil, and other plants with poor root systems were grafted on the wild-type seedling, which grew in the greenhouse.

*Gossypium hirsutum* cv. CCR35, transgenic plants and transgenic offspring were grown in a greenhouse under 28°C and 14/10 h light/dark conditions. The T0 transgenic plants (CRISPR/Cas9 editing mutants) in the greenhouse were self-pollinated, and the seeds were finally collected. Due to the abnormal phenotypes in the T0 plants with stunted growth and fewer flowers, we acquired an 11 to 64 seeds from the 16 *Gh14-3-3d*-edited plants. For genetic analysis of the mutant genotypes, T1 plants were then planted under natural conditions in Yuncheng, Shanxi province, China (N35°04′, E110°58^′^).

### CRISPR/Cas9 Vector Construction and Selection of sgRNA Targets

Based on simplifying the assembly process of the target sequence of sgRNAs, the sequence of AtU3b-double *Bsa*I-sgRNA (**Supplementary Data S1**) was synthesized, then cloned into pYLCRISPR/Cas9-N through *Bsa*I sites (Ma et al., 2015), resulting in vector designated as pYLCRISPR/Cas9-NDB.

The sgRNA targets were predicted using the online tool Cas-Designer based on the *G. hirsutum* database (Bae et al., 2014). Among these target sites, a target site in exon1 with higher scores that simultaneously targeted the two copies of the *Gh14-3-3d* gene, *Gh14-3-3d-A* and *Gh14-3-3d-D* located in the allotetraploid cotton At and Dt subgenome, respectively, was selected. The target sequence (AGATGAAGGGAGATTACCAT) was directly inserted into pYLCRISPR/Cas9-NDB by double *Bsa*I to obtain the Gh14-3-3d editing vector, pYLCRISPR/Cas9-NC14.

### VIGS Mediated by Agrobacterium

A specific *Gh14-3-3d* fragment was amplified by PCR and directly cloned into the tobacco rattle virus (TRV) vector pYL-156, a gift from Professor Yule Liu at Tsinghua University. The resulting vector was named pYL-156-Gh14-3-3d. The pYL-156-Gh14-3-3d, pYL-156 (empty vector) and the pYL-192 helper vector were transformed into the *Agrobacterium tumefaciens* GV3101 strains by electroporation.

Agrobacteria culture and inoculation solution preparation were carried out according to a previous report (Wang L. et al., 2017). Agrobacteria containing pYL-156-Gh14-3-3d or pYL-156 were equally mixed with pYL-192, and injected into fully expanded cotyledons using a needleless syringe. After 14 days, the plants injected with Agrobacteria were inoculated with *V. dahliae* for defense analysis.

### DNA Extraction, PCR Reaction, and Sequencing Assay

Genomic DNA of the cotton leaves was extracted using the Plant Genome Extraction Kit (TianGen, Beijing, China) according to the instruction manual. PCR was performed to detect the T-DNA components and monitor the indels of the *Gh14-3-3d* genes in transgenic plants and their offspring. *Cas9, Npt II*, and three sequence fragments, including *Npt II-nos-ter, p35s*-*Cas9*, and *sgRNA* expression cassette, were analyzed by common PCR methods, whose specific primers were listed in **Supplementary Table S1**.

To amplify the genomic region surrounding the CRISPR target sites, two rounds of PCR reactions were performed in independent transgenic cotton plants and their offspring. In the first round of PCR, the two specific PCR fragments of *Gh14-3-3d-A* (1984 bp) and *Gh14-3-3d-D* (1957 bp) were amplified from the genomic DNA by a special forward primer located in the At or Dt subgenomes (PA or PD) and a universal reverse primer (PR). In the second round of PCR, a specific fragment of approximately 620 bp with a target site was acquired through a pair of universal primers (PF and PR) when the specific PCR fragment of the At or Dt subgenomes from the first-round PCR products was employed as a PCR template. To identify the indels of the mutants, these PCR products were directly sequenced, or cloned into the pEASY-T3 (TransGen) vector and then sequenced by Sanger method.

Fungal biomass quantification was performed through comparing the *V. dahliae β-tubulin* DNA levels to the cotton *UB7* DNA levels after *V. dahliae* inoculation by qPCR according to the method described previously (Atallah et al., 2007; Wang L. et al., 2017). The relative primers were shown in **Supplementary Table S1**. The same experiment was carried out using three biological replicates.

### T7EI Assay for Mutation Identification

The second-round PCR products of each sample mentioned above, a 620 bp fragment with target sites, were equivalently mixed with the PCR fragments amplified from the WT plant, which were used to detect the mutation with T7 Endonuclease I (Vazyme, Nanjing, China) according to the instruction manual. Final digested reaction products were analyzed with 1.5% agarose gel electrophoresis.

### RNA Isolation, Reverse Transcription, and Real-Time PCR

Total RNA of the cotton leaves and roots was isolated using the Plant Total RNA Extraction Kit (Sangon Biotech, Shanghai, China) according to the instruction manual. Two micrograms of RNA was reverse transcribed for first strand cDNA synthesized following the manufacturer’s protocol using the TransScript First-Strand cDNA Synthesis kit (TransGen).

According to the protocols of the Minimum Information for Publication of Quantitative Real Time PCR Experiments (Bustin et al., 2009), diluted cDNA was used for qPCR with SYBR green using the CFX96 Touch^TM^ Real-Time PCR detection systems (Bio-Rad, Foster City, CA, United States). All gene expression was calculated using the dCt or ddCt algorithm. To normalize the gene expression, the *UB7* gene was used as an internal standard. All the gene specific primers involved in this study were listed in **Supplementary Table S1**.

### Pathogen Culture and Inoculation

The defoliating isolate V991 of *V. dahliae* was cultured on potato dextrose agar for 3 days, and then the fungus was incubated in Czapek’s medium (sucrose, 3% w/v; NaNO_3_, 0.3% w/v; KCl, 0.1% w/v; KH_2_PO_4_, 0.1% w/v; MgSO_4_, 0.1% w/v; FeSO_4_, 0.0002% w/v; pH 6.0) and grown in the dark at 25°C for 5 days. The spore concentration of the fungus was adjusted to 10^5^ conidia/ml with 5% deionized sucrose solution for inoculation. The 21-day-old seedlings were inoculated with *V. dahliae* or sucrose solution (mock) through the roots. The inoculated seedlings were incubated in a chamber at 25°C under a 14/10 h light/dark photoperiod.

To evaluate plant resistance to *V. dahliae*, the rate of disease in plants and the disease index (DI) of the plants were investigated according to the methods reported by Wang L. et al. (2017). Additionally, a fungal recovery experiment was performed 14 days after infection according to the method described previously (Wang L. et al., 2017). The same experiment was carried out using three biological replicates.

## Results

### Sequence Identification for Two Copies of the *Gh14-3-3d* Targeting Gene and Assembly of sgRNAs

**FIGURE 1:**
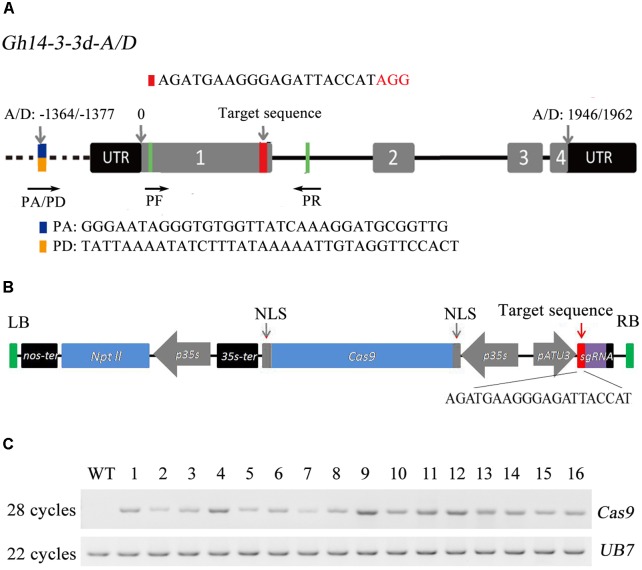
The structure of the *Gh14-3-3d* gene, editing vector construction and PCR analyses of the transgenic plants. **(A)** The structure of *Gh14-3-3d-A* and *Gh14-3-3d-D* (*Gh14-3-3dA/D*) and the target site of the sgRNA in exon1. The target site was highlighted in red, the difference between sequences of *Gh14-3-3dA/D* are labeled in blue-yellow (special forward primers, PA/PD, were designed in this site), and the universal primer sites (PF and PR) were highlighted in green. The AGG in red represents the PAM motif. The different numbers of nucleotides noted by arrows indicated different sites, start codons and stop codon. **(B)** The schematic of the T-DNA region of the editing vector pYLCRISPR/Cas9-NC14. The target sequence of the *Gh14-3-3dA/D* genes was listed under the schematic. **(C)** RT-PCR analysis of the *Cas9* transcript level in the WT and transgenic plants. The *UB7* gene was regarded as the inner control. WT, wild-type plant; lanes labeled 1 to 16 represent the 16 independent transformants.

To simplify the assembly process of the target sgRNA sequences, we constructed a CRISPR/Cas9 toolkit with double *Bsa*I for easy and convenient insertion of target sequences in plant target genes or DNA editing (**Supplementary Figure S1**). A previous study has shown that 14-3-3d is a negative regulator in the BR signal pathway that participates in cotton defense. In this study, the *Gh14-3-3d*-silenced plants by VIGS were produced to confirm its function in defense against *V. dahliae*. As shown in **Supplementary Figure S2**, the *Gh14-3-3d*-silenced plants showed a higher resistance compared to the wild-type plants. Thus, the *14-3-3d* gene was chosen and edited for increasing cotton plant defense to *V. dahliae* by CRISPR/Cas9-mediated mutations. The *Gh14-3-3d* gene has two copies located in the tetraploid cotton At and Dt subgenomes (GenBank accession Nos. NM_001327374 and XM_016860147), which were referred to as *Gh14-3-3d-A* and *Gh14-3-3d-D*. Both share a highly similar nucleotide sequence and the same gene structure (**Figure 1A**). The two copies of this gene encode proteins that share 98.9% identification in amino acids and there are just three different sites among 261 amino acids (**Supplementary Figure S3**). We chose an excellent target site with 20-bp followed by the AGG PAM motif located in exon1 downstream of the *Gh14-3-3d* gene (**Figure 1A**), as sgRNA targeted both *Gh14-3-3d-A* and *Gh14-3-3d-D*. For designing the CRISPR/Cas9 vector, a 20-bp target sequence of *Gh14-3-3d* was inserted directly in the vector mentioned above by *Bsa*I digestion and driven by *AtU3b* promoter (**Figure 1B**).

### Evaluation of CRISPR/Cas9-Mediated Mutagenesis of *Gh14-3-3d* in T0

It took 1 year to perform cotton genetic transformation, and we acquired 31 regenerating plants with kanamycin resistance that could grow in the greenhouse (**Supplementary Figure S4**). Among these plants, 16 plants with *Cas9* gene was successfully expressed in T0 plants by RT-PCR analysis (**Figure 1C**). Finally, the 16 T0 plants were subject to analyses of nucleotide insertion and deletion mediated by the CRISPR/Cas9 genome editing system.

**FIGURE 2:**
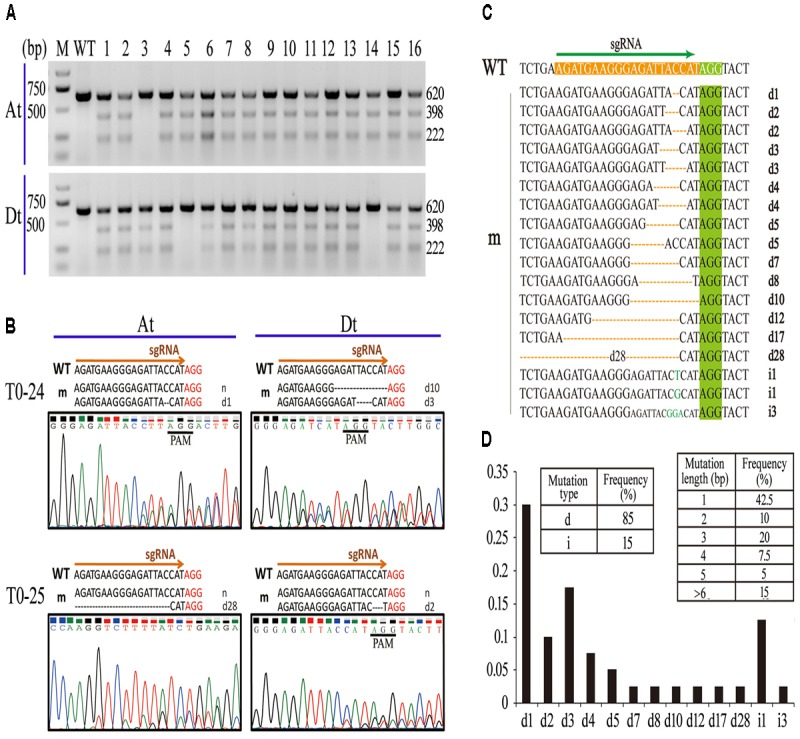
Analyses of CRISPR/Cas9-mediated mutagenesis of Gh14-3-3d in T0 plants. **(A)** T7EI digestion assay of the mutants at the target sites. The sizes (bp) of the digested amplifications for the *Gh14-3-3d* at At (Top) or Dt (Bottom) were indicated on the right side. Each amplification from the transgenic plants was equally mixed with the PCR product of the WT. M, DNA molecular marker; WT, wild-type plant; lanes labeled 1 to 16 represent the 16 independent transformants (mutants) mentioned above. **(B)** The indels of *Gh14-3-3d* at the target site of the At or Dt subgenome in T0–24 and T0–25 were illustrated based on the nucleotides and chromatograms by Sanger sequencing, respectively. The PAM sequence was shown in red. The detailed indels labeled at sequence right. d#, # of base pairs (bp) deleted from the target site; i#, # of base pair (bp) inserted at the target site, which was used thereafter. **(C)** Different indels of *Gh14-3-3dA/D* occurred at expected target site in the 16 mutants. The PAM motif was highlighted in green. The indel types are listed at the right. WT, the wild type; m, mutants. **(D)** The indel types and frequency in the 16 mutants. d, deletion; i, insertion.

PCR products of the At and Dt subgenomes from 16 transgenic plants in T0 were acquired using two pairs of special primers (listed in **Supplementary Table S1**) through two cycles of PCR amplification and then were cleaved by the T7EI digestion to examine whether the *Gh14-3-3d-A* and *Gh14-3-3d-D* of these T0 plants were edited by the CRISPR/Cas9 system. Most of the PCR fragments were cleaved into two parts at the expected target site, indicating that two copies of the *Gh14-3-3d* gene were edited, generating indels at the expected target site (**Figure 2A**). Notably, if the PCR amplification of the T0 plants was obtained by a pair of common primers for the At and Dt subgenomes, they could be cleaved into lots of parts by the T7EI digestion due to sequence differences of the two copies of *Gh14-3-3d* (**Supplementary Figure S5A**). Thus, it was necessary to distinguish from different copies of polyploidy plants for employing the T7EI digestion to identify mutagenesis by the CRISPR/Cas9 genome editing system.

To intensively explore the generated indels, the PCR products were directly sequenced by Sanger sequencing. Because *G. hirsutum* is an allotetraploid cotton species, PCR products of 16 T0 plants acquired using a common primer pair showed a notable phenomenon in the sequencing chromatogram. As shown in **Supplementary Figure S5B**, a typical sequencing chromatogram for these PCR fragments presented single peaks extending up to the mutation sites of the sgRNA target sequence, but immediately after the mutation sites multiple peaks often started to appear in each nucleotide position. Although Sanger sequencing chromatograms can be read by an artificial method and special program (**Supplementary Figure S5B**), it was unclear if these indels occurred at *Gh14-3-3d-A* or *Gh14-3-3d-D*. For distinguishing these indels from the At or Dt subgenomes, the PCR fragments amplified by two pairs of special primers were sequenced. The results showed 1–2 types of indels at the expected target site in the At or Dt subgenomes, so there were 1–4 types of indels in each plant mediated by CRISPR/Cas9 system (**Table 1** and **Figure 2B**). For instance, T0–30 transgenic plants presented 4 types of indels, d1d3d5i3 (1 and 3 bp deletions at two alleles of At subgenome, 5 bp deletion and 3 bp insertion at two alleles of Dt subgenome), while T0–6 and T0–9 containing 1 indel, d1 at the Dt subgenome and d3 at the At subgenome, respectively. Other plants possess 2–3 types of indels at the target site. According to the genotype analysis, there were some homozygote or bi-allele indels at *Gh14-3-3d-A* or *Gh14-3-3d-D*, but only 2 plants, T0–12 and T0–30, possessed homozygote or bi-allele indels at both the At and Dt genomes (**Table 1**).

**Table 1:** Mutation genotypes in independent *Gh14-3-3d* transgenic T0 plants.

	A subgenome		D subgenome	
Plants	Zygosity	Genotype	Zygosity	Genotype
T0–1	Heterozygote	WT d2	Heterozygote	WT d4
T0–3	Heterozygote	WT d1	Bi-allele	d3^∗^ i1
T0–6	/	WT WT	Heterozygote	WT d1
T0–8	Bi-allele	d1 d17	Heterozygote	WT d4
T0–9	Heterozygote	WT d3	/	WT WT
T0–12	Homozygote	d1 d1	Bi-allele	d3 i1
T0–15	Heterozygote	WT d2	Homozygote	WT d1
T0–16	Bi-allele	d3 d1	Heterozygote	i1 WT
T0–17	Bi-allele	WT i1	Heterozygote	d1 d8
T0–20	Heterozygote	WT d2	Heterozygote	WT d7
T0–22	Heterozygote	WT d4^∗^	Heterozygote	WT d1
T0–24	Heterozygote	WT d1	Bi-allele	d10 d3
T0–25	Heterozygote	WT d28	Heterozygote	WT d2^∗^
T0–27	Bi-allele	d5 i1^∗^	/	WT WT
T0–28	Heterozygote	WT d3	Bi-allele	d1 d12
T0–30	Bi-allele	d5^∗^ i3	Bi-allele	d1 d3

*^∗^Different mutation sites with same bp number of indels.*

To precisely analyze these indels of each allele at both the At and Dt genomes, more than 150 positive colonies from PCR amplification of the edited *Gh14-3-3d-A* or *Gh14-3-3d-D* in the 16 T0 plants were randomly picked for the sequencing analysis. The result showed that 40 editing events independently occurred at the At or Dt subgenomes of 16 plants. Eighteen of these sequences showed different indels at the expected target site, suggesting that many types of genome editing events precisely occurred at the examined target gene, *Gh14-3-3d* (**Figure 2C** and **Table 1**). These indels were randomly presented at the target site of *Gh14-3-3d-A* or *Gh14-3-3d-D*, with 11 types of indels at both the At and Dt subgenome (**Table 1**). The results of these editing sequences showed that 35 of 40 editing events were nucleotide deletions and the others were insertion events, which suggested that deletions were more common than insertions in the cotton CRISPR/Cas9 genome editing system (**Figure 2D**). Among the 40 editing events, 12 were 1 bp nucleotide deletions, 5 exhibited 1 bp nucleotide insertions, indicating that 1 bp nucleotide indels mutants were readily generated during CRISPR/Cas9-mediated genome editing in cotton (**Figure 2D**). These results indicated that the sgRNA targeted *Gh14-3-3d* genes effectively and precisely guided Cas9-mediated genome cleavage, resulting in a highly effected target sequence mutant including nucleotide deletion and insertion. Thus, this CRISPR/Cas9 genome editing system has a high potential for producing different indel mutants on the tetraploid cotton genome for improving the cotton cultivars.

### Evaluation of CRISPR/Cas9-Mediated Mutagenesis of *Gh14-3-3d* in T1

For clarifying the stabilization and genetic pattern of these indels at the At and Dt subgenomes of tetraploid cotton, the mutated sequence in editing mutants and their offsprings were analyzed. In T0, few of the seeds from the 16 *Gh14-3-3d*-edited plants were harvested because of the stunted growth and fewer flowers of the regenerated plants. T1 plants were then planted under natural conditions for genetic analysis of the mutant genotypes in Yuncheng, Shanxi province, China. The results of the PCR analysis suggested that the segregation ratios of the Cas9 gene mostly followed Mendelian laws in the 16 T1 lines, possibly presenting a 3:1 segregation ratio in T0–1, 6, 15, 20, 22, and 24 lines (**Table 2**). More importantly, the genetic patterns of the indels in the 16 T1 lines were analyzed by PCR and sequencing approaches to investigate the mutant genotype segregation of the At and Dt subgenome in the offspring. All indels generated in the T0 plants can stably transmit to T1 plants, which mostly met the genetic laws in the At and Dt subgenomes (**Tables 1**, **2**). Interestingly, we found that there was a new indel (d3 in the At subgenome) at the target site in an offspring plant of the T0–6, indicating that Cas9 functioned in offspring plants to produce novel target gene editing (**Table 2** and **Supplementary Figure S6**). As shown in **Table 2**, the *Gh14-3-3d* gene indels in the T1 lines showed independent segregation in T-DNA insertion, resulting in a few of mutant plants whose T-DNA were segregated and had already been eliminated. Thus, we may easily choose mutagenized plants free of T-DNA from these T1 lines.

**Table 2:** Segregation of mutation genotypes in *Gh14-3-3d-*edited T1 lines.

Lines	T0 plant genotype	No. of plants with Cas9+/Cas9-	Segregation ratio in T1 lines	No. of homozygous plants with Cas9+ or Cas9-
T0–1	WTd2WTd4	18/4^$^	12WT_WT_, 4d2d2WT_, 5WT_d4d4, 1d2d2d4d4	1d2d2d4d4/Cas9+
T0–3	WTd1d3i1	17/0	5WT_d3d3, 8WT_d3i1, 2WT_i1i1, 1d1d1d3d3, 1d1d1d3i1	1d1d1d3d3/Cas9+, 1d1d1d3i1//Cas9+
T0–6	WTWTWTd1	13/3	13WTWTWT_, 3WTWTd1d1, 1WTd3WTWT^&^	/
T0–8	d1d17WTd1	7/1	2WT_d1d1, 3WT_d1d17, 1WT_d17d17, 1d1d1d1d17	1d1d17d17/Cas+
T0–9	WTd3WTWT	9/1	8 WT_WTWT, 2d3d3WTWT	/
T0–12	d1d1d3i1	7/0	2d1d1d3d3, 5d1d1d3i1	2d1d1d3d3/Cas+, 5d1d1d3i1/Cas+
T0–15	WTd2WTd1	17/5	12WT_WT_, 5d2d2WT_, 6WT_d1d1, 2d2d2d1d1	1d2d2d1d1/Cas+, 1d2d2d1d1/Cas-^∗^
T0–16	d3d1WTi1	15/0	2d1d1WT_, 6d3d1WT_, 4d3d3WT_, 2d3d1i1i1, d1d1i1i1	2d3d1i1i1/Cas+, d1d1i1i1/Cas+
T0–17	WTi1d1d8	15/1	4WT_d1d1, 7WT_d1d8, 2WT_d8d8, 1i1i1d1d8, 1i1i1d8d8	1i1i1d1d8/Cas+, 1i1i1d8d8/Cas+
T0–20	WTd2WTd7	11/4	8WT_WT_, 4d2d2WT_, 3WT_d7d7	/
T0–22	WTd4WTd1	16/3	10WT_WT_, 5d4d4WT_, 4WT_d1d1	/
T0–24	WTd1d10d3	17/7	5WT_d10d10, 11WT_d10d3, 4WT_d3d3, 2d1d1d10d10, 2d1d1d10d3	1d1d1d10d10/Cas+, 1d1d1d10d10/Cas-^∗^, 2d1d1d10d3/Cas+
T0–25	WTd28WTd2	15/1	11WT_WT_, 2d28d28WT_, 3WT_d2d2	/
T0–27	d5i1WTWT	21/1	11WTWTd5i1, 5WTWTi1i1, 6WTWTd5d5	/
T0–28	WTd3d1 d12	15/0	4WT_d1d1, 7WT_d1d12, 2WT_d12d12, 2d3d3d1d12	2d3d3d1d12/Cas+
T0–30	d5i3d1d3	6/0	3d5d5d1d3, 1d5i3d1d1, 2d5i3d1d3	3d5d5d1d3/Cas+, 1d5i3d1d1/Cas+, 2d5i3d1d3/Cas+

^$^All seeds harvested from T0 were planted; ^&^a new indel taken place in T1 editing line; ^∗^the transgene-clean *Gh14-3-3d*-edited plant; WT_, represents WTWT, WTd#, or WTi#.

### The Identification of the Transgene-Clean Editing Lines With Homozygous Mutagenesis of *Gh14-3-3d*

**FIGURE 3:**
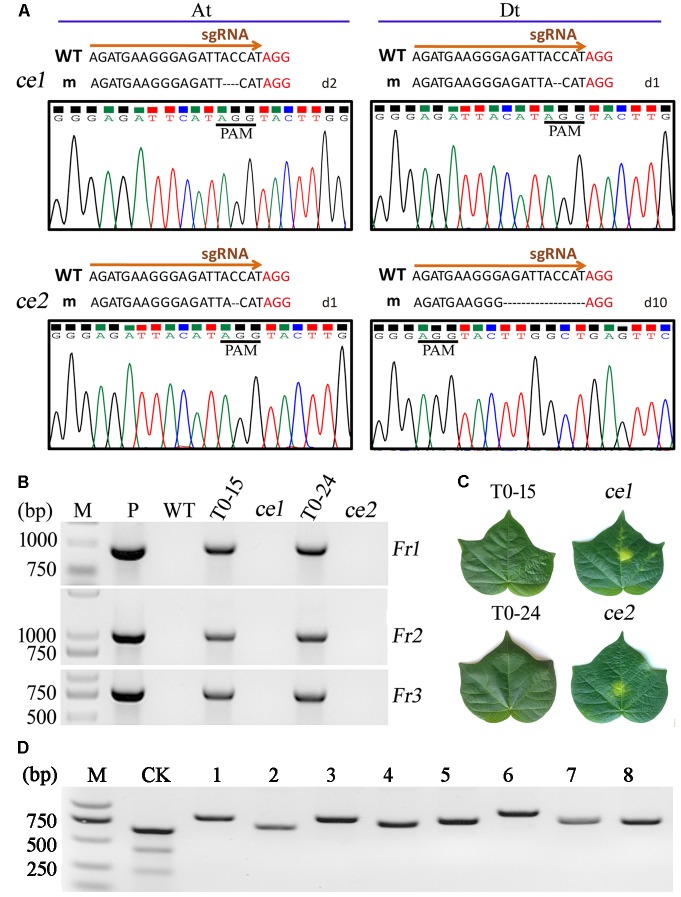
Analyses of the two transgene-clean editing lines with homozygous mutagenesis of *Gh14-3-3d.* **(A)** The two transgene-clean editing lines, *ce1* and *ce2*, exhibited indels at the target site of the At or Dt subgenome based on the nucleotides and chromatograms by Sanger sequencing, respectively. The PAM sequence was shown in red. **(B)** The T-DNA free in the *ce1* and *ce2* plants detected by PCR analysis. M, DNA molecular marker; P, plasmid vector as a positive control; WT, wild-type plant. The *ce1* and *ce2* were segregated from the T0–15 and T0–24 transformants, respectively. Fr1, Fr2, and Fr3 were fragments of the *Npt II-nos-ter* (912 bp), *p35s*-*Cas9* (1012 bp), and *sgRNA* expression cassettes (725 bp), respectively. **(C)** The leaf parts painted with 0.5% kanamycin solution were yellow in the *ce1* and *ce2* plants, while they were still green in the T0–15 and T0–24 plants. Photographs of the leaves were taken at 7 days after painting. **(D)** The PCR products from eight genes containing putative off-target sequences with 1–3 bp mismatches in *ce1* and *ce2* were not cleaved by T7EI.

To evaluate the function of *Gh14-3-3d*, the mutant plants with homozygous or bi-alleles in the tetraploid cotton At and Dt subgenomes were screened. As shown in **Table 2**, 10 lines with homozygous or bi-allele indels were found in the T1 segregation groups. Considering the safety of transgenic plants, the T-DNA of the *Gh14-3-3d*-edited plants must be ruled out, which were called transgene-clean editing target gene plants. There were 2 transgene-clean plants with homozygous *Gh14-3-3d* editing that were segregated from the T0–15 and T0–24 lines, whose genotypes were d2d2d1d1 and d1d1d10d10, respectively, designated *ce1* and *ce2* (**Table 2** and **Figure 3A**). To confirm whether the *ce1* and *ce2* lines contained transgenic components, the three fragments of the T-DNA from LB to RB, including the *Npt II-nos-ter* (912 bp), *p35s*-*Cas9* (1012 bp), and *sgRNA* expression cassettes (725 bp), were amplified by PCR. The results showed that no amplifications were present in the two transgene-clean plants and WT plants, while the three corresponding fragments as well as the *Cas9* gene were amplified in the transgenic plants shown in **Table 2**, suggesting that the T-DNA had been ruled out in two transgene-clean plants (**Figure 3B**). Additionally, the result of the kanamycin-resistance assay showed that the leaf dots of the transgene-clean plants painted with 0.5% kanamycin solution became yellow 5 days later, while the transgene leaves were still green (**Figure 3C**). To investigate the genetic stability of the indels in *ce1* and *ce2*, 20 offspring plants (T2) were used to carry out PCR and sequencing analyses. The results showed that the T2 plants contained the same indels as *ce1* or *ce2* mutagenesis (**Figure 3A**), showing d2d2d1d1 or d1d1d10d10 in the genotypes, respectively, suggesting that *Gh14-3-3d* editing genotypes were stably transmitted into the next generation at the At and Dt subgenomes, and there were no novel indels generated in the offspring plants. Altogether, these results showed that the *ce1* and *ce2* lines were successfully developed through the CRISPR/Cas9-targeted genome editing system.

### Potential Off-Target Analysis in the Two Transgene-Clean Editing Lines

To evaluate the off-target potential in *ce1* and *ce2* to affect other phenotypes, we analyzed the off-target effects of the putative off-target sequences obtained through blasting the cotton genome database^1^. These potential off-target sites contained 14 bp mismatches compared to the on-target guide sequences of *Gh14-3-3d* gene as shown in **Supplementary Table S2**. The eight putative off-target sequences with 1–3 bp mismatches located in genes were selected for further analysis. In *ce1* and *ce2*, the PCR products from eight genes containing putative off-target sequences were not cleaved by T7EI, which was unlike the results in **Figure 2A**, showing the novel cleaved fragments (**Figure 3D**). The sequencing results of the PCR amplifications showed that there were no differences among the sequences of potential off-target sites in *ce1, ce2*, and WT plants, indicating that no editing was detected in these putative off-target sites (**Supplementary Table S2**). Additionally, the results of Sanger sequencing showed that putative off-target sequences of eight genes from the cotton gene database were the same as real sequences of the potential off-target sites by PCR in *ce1, ce2*, and WT plants. Those data showed that no mutations were observed in the examined putative off-target genes of *ce1* and *ce2* plants, possibly indicating that of the CRISPR/Cas9 genome editing toolkit has high specificity in plants.

### Transgene-Clean Editing *Gh14-3-3d ce1* and *ce2* Enhance Resistance to *V. dahliae*

**FIGURE 4:**
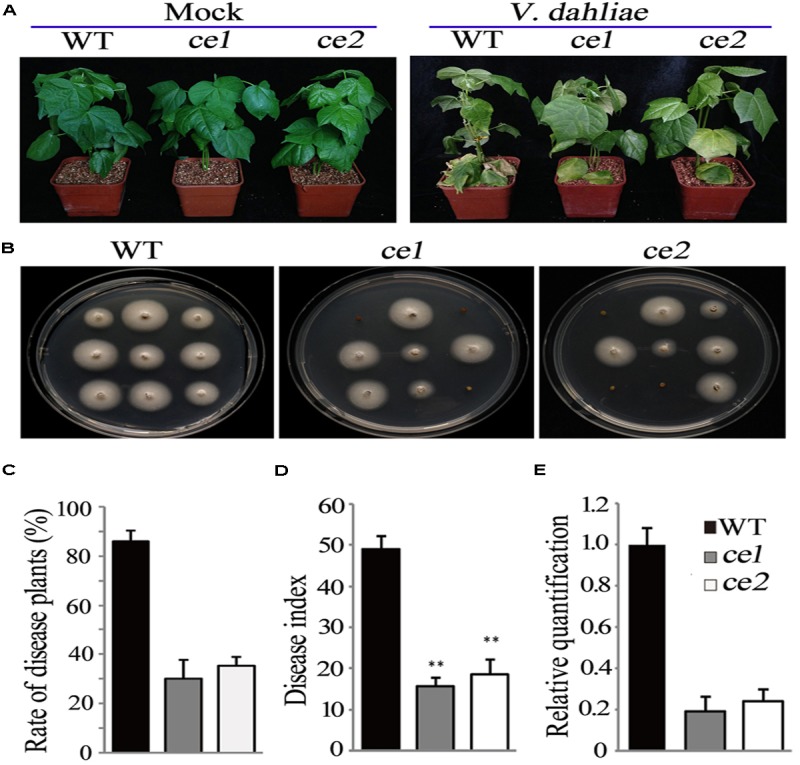
Disease symptom analysis of the *ce1* and *ce2* lines inoculated with *V. dahliae.* **(A)** Photographs of the representative WT, *ce1* and *ce2* plants taken at 18 days after inoculation with *V. dahliae* (10^5^ conidia/ml) or mock. **(B)** Fungal recovery assay. The stem sections of the plants inoculated with *V. dahliae* (10^5^ conidia/ml) 18 days later were placed on potato dextrose agar medium, and then photographs were taken 4 days after culture. **(C,D)** The rate of diseased plants and disease index in the WT, *ce1*, and *ce2* plants. Error bars represent the SD of three biological replicates (*n* ≥ 30). Double asterisks indicate statistically significant differences compared to the control, which were analyzed using Student’s *t*-test (*P* < 0.01). **(E)** The relative quantification of the fungal biomass of the cotton leaves among the WT, *ce1*, and *ce2* plants by qPCR. In comparing the *V. dahliae β-tubulin* gene DNA levels to the cotton *UB7* DNA levels 18 days after *V*. *dahliae* inoculation, the data in the WT was normalized as “1.” Error bars represent the standard deviation of the three biological replicates.

To examine the resistance of the transgene-clean editing lines with *Gh14-3-3d* mutations, 21-days seedlings of *ce1* and *ce2* were infected by *V. dahliae* along with the WT plants. At 18 days after inoculation, two transgene-clean editing lines and the WT plants healthy grew under the treatment with mock, while both had increased resistance to *V. dahliae* with less severe defoliation and yellowing symptoms compared to the WT plants (**Figure 4A**). A fungal recovery assay was performed to further investigate the response of *ce1* and *ce2* plants to *V. dahliae* infestation. The result showed that obviously fewer stem sections from the transgene-clean editing plants grew mycelium compared to the WT (**Figure 4B**). The disease plant rate and disease index in the *Gh14-3-3d*-edited plants was significantly lower than those in WT (**Figures 4C,D**). Additionally, the fungal biomass of the leaves from the *ce1* and *ce2* plants at 18 days after infection obviously decreased compared to that of the WT as determined by qRT-PCR analysis, just reaching 0.19- and 0.22-fold of the WT, respectively (**Figure 4E**). Taken together, the data suggested that the two transgene-clean *Gh14-3-3d*-edited lines showed higher resistance to *V. dahliae*.

**FIGURE 5:**
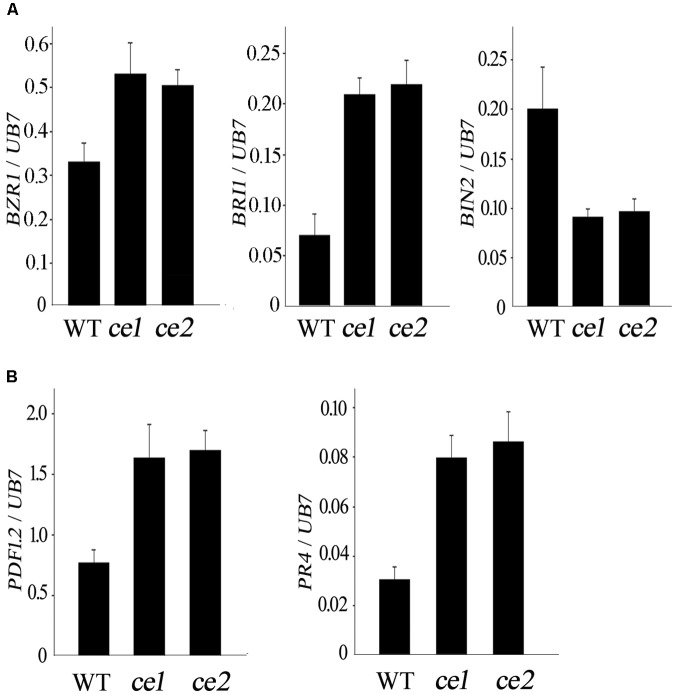
Expression patterns of the three BR signal genes **(A)** and two JA defense-related marker genes **(B)** in the WT, *ce1*, and *ce2* plants treated with *V. dahliae*.

To explore whether the enhanced resistance to *V. dahliae* in *Gh14-3-3d*-edited plants was involved in the BR signal pathway and defense-related marker genes, the expression levels of *Brassinosteroid insensitive 1* (*BRI1*), *BZR1, BIN2, PDF1.2*, and *PR4* were monitored by qRT-PCR analysis. The expression pattern of the three BR signal genes in the roots of *ce1* and *ce2* inoculated with *V. dahliae* showed significant differences compared to the WT, with *BRI1* and *BZR1* exhibiting up-regulated expression and *BIN2* showing down-regulated expression (**Figure 5A**), suggesting that Gh14-3-d-3d participated in plant defense against *V. dahliae* possibly through the BR signal pathway. Most of the genes involved in JA signaling were up-regulated in cotton plants after treatment of BR, but there were no obvious changes in the transcripts of the SA signaling pathway-related genes (Gao et al., 2013). Thus, we examined the expression levels of *PDF1.2* and *PR4*, two well-known JA-regulated defense-related marker genes; both significantly up-regulated expression in the two transgene-clean *Gh14-3-3d*-edited lines infected with *V. dahliae*, showing nearly twofold and threefold higher of the WT, respectively (**Figure 5B**). The result suggested that the *ce1* and *ce2* lines possessed higher resistance to *V. dahliae*, possibly by modulating BR and JA signaling gene expression.

## Discussion

The CRISPR/Cas9 genome editing system had been developed in *G. hirsutum* as determined by editing endogenous and exogenous marker genes (Chen et al., 2017; Janga et al., 2017; Li C. et al., 2017; Wang P. et al., 2017). Thus, the interesting and economical genes edited by the CRISPR/Cas9 technique remained for the study of improving cotton cultivars. In this study, lots of *Gh14-3-3d* indels were generated in the tetraploid cotton At and Dt subgenomes mediated by our CRISPR/Cas9 genome editing toolkit. More importantly, the transgene-clean T2 lines with homozygous editing mutagenesis of *Gh14-3-3d* were developed, which possessed high resistance to *V. dahliae* infestation.

We employed the CRISPR/Cas9-targeted gene editing system to generate lots of indels of the *Gh14-3-3d* gene in the tetraploid cotton At and Dt subgenomes, and successfully bred the homozygous *Gh14-3-3d*-edited plants. Lots of plant genomes are polyploid in nature, which leads to high production and defense potential, such as allotetraploid cotton, tetraploid potato, hexaploidy wheat, and so on. In general, it is difficult for polyploid plants to generate mutants by natural mutation and artificial mutation due to the functional redundancy of multiple copies of genes (Wang Y. et al., 2014; Braatz et al., 2017; Lu et al., 2017; Meng et al., 2017). Now it is feasible to produce target gene mutants by CRISPR/Cas9 genome editing system. For example, Wang Y. et al. (2014) reported that the three copies of the *TaMLO* gene were simultaneously edited in a plant, resulting in increasing resistance to powdery mildew. Similar study in wheat was performed through editing three homeologs of *TaEDR1* to increase the resistance to powdery mildew (Zhang et al., 2017). Three different regions of the gene encoding granule-bound starch synthase were targeted to change the starch in the tetraploid potato using the CRISPR/Cas9 technique (Andersson et al., 2017). Braatz et al. (2017) employed the CRISPR/Cas9 genome editing system to target two *BnALC* homeologs, resulting in reducing seed shattering in the mutant of oilseed.

Plant 14-3-3 proteins play important roles in development and defense through kinases, phosphatases and member acceptors (Obsil et al., 2001; Gao et al., 2013). In cotton, 14-3-3 proteins mainly participate in cotton fiber development (Shi et al., 2007; Zhang et al., 2010; Zhou et al., 2015) and defense (Hill et al., 1999; Yan et al., 2004; Sun et al., 2011). For instance, three Gh14-3-3 family proteins, Gh14-3-3e, Gh14-3-3h, and Gh14-3-3L, promoted fiber initiation and elongation (Zhou et al., 2015). Gao et al. (2013) showed that Gb14-3-3c and Gb14-3-3d negatively regulate cotton plant resistance to *V. dahliae* through participating in the BR signaling pathway. Notably, these studies on cotton 14-3-3 protein function mostly depended on regulating RNA transcript levels by RNAi and overexpression approaches. In this study, we developed two transgene-clean *Gh14-3-3d*-edited lines, *ce1* and *ce2*, using the CRISPR/Cas9 genome editing system. Both mutant lines had increasing resistance against *V. dahliae* infestation and can be used as a germplasm to breed cotton disease-resistant cultivars. Notably, fiber length of the mutants *ce1* and *ce2* was comparable with the WT possibly due to *Gh14-3-3d* specially responding to defense, which was consistent with the result in *G*. *barbadense* (Gao et al., 2013).

In the past 20 years, application of transgenic cotton, including insect- and herbicide-resistant cultivars, has brought large economic benefits, such as 1.5 billion dollars in increased income due to the Bt cotton plant each year in China (Li and Zhang, 2016). However, before the transgenic plants grow in the open environment, a strict safety assessment must be carried out according to the policies and regulations issued by the countries or organizations. The safety assessment of the transgene crops is a time-consuming, expensive and complex process. Thus, it is a pivotal to breed transgene-clean editing plants from transgenic lines with target gene editing. Recently, the development of transgene-clean editing plants emerged in crops, including soybean, oilseed rape and wheat (Braatz et al., 2017; Cai et al., 2017; Li R. et al., 2017; Zhang et al., 2017). We developed transgene-clean cotton lines by editing *Gh14-3-3d*, which can highly resist *V. dahliae* infestation.

## Conclusion

Lots of *Gh14-3-3d* indels in editing plants were identified by PCR and sequencing analyses. These indels in tetraploid cotton At and Dt subgenomes could be stably transmitted into the next generation and were segregated in T1 populations according to Mendelian laws. The two transgene-clean editing plants with homozygous mutations, *ce1* and *ce2*, were produced in T1. The *Gh14-3-3d*-edited plants in T2 showed a higher resistance to *V. dahliae* compared to the wild-type plants. The two transgene-clean lines were directly used as germplasms to breed defense cultivars, which could be free of the safety assessments for transgenic crops.

## Author Contributions

JW and FL conceived and designed the experiments. ZZ and XL performed the experiments. PW, QF, GH, and JX constructed the gene editing vectors and data analysis. XG and JW wrote the paper. All authors read and approved the final manuscript.

## Conflict of Interest Statement

The authors declare that the research was conducted in the absence of any commercial or financial relationships that could be construed as a potential conflict of interest.

## Footnotes

1 https://www.cottongen.org/species/

## Supplementary Material

FIGURE S1 Schematic of the T-DNA region of plant gene editing vector pYLCRISPR/Cas9-CBD. The sequence of *Bsa*I recognition motifs were highlighted in yellow, digestion site of *Bsa*I were shown by red lines.

FIGURE S2 Increased resistance of the Gh14-3-3d-silenced plants to *V. dahliae*. **(A)** *Gh14-3-3d* expression levels in silenced plants (*TRV: Gh14-3-3d*) and the control (*TRV: 00*) were determined by qPCR. The *Gh14-3-3d* expression level of the control was designated 1, and the average expression level in *Gh14-3-3d* -silenced plants was determined from 15 independent tested plants (3 replicates and 5 plants per replicate). **(B)** Disease symptoms on *Gb14-3-3d-*silenced plants and the control at 10th day post-inoculated with *V. dahliae.* **(C)** The disease index of the *Gh14-3-3d*-silenced plants and the control. Error bars represent the SD of three biological replicates (*n* ≥ 36). Asterisk indicates statistically significant differences compared to the control using Student’s *t*-test (*P* < 0.05).

FIGURE S3 Amino acid alignment of the two copies of Gh14-3-3d encoding proteins, Gh14-3-3d-A and Gh14-3-3d-D.

FIGURE S4 The putative transgenic plants in T0 in greenhouse. The regenerate plantlets were directly transplanted, or grafted on receptor seedlings in pots with 1:3 vermiculite and organic matter soil.

FIGURE S5 Two copies of Gh14-3-3d affect the analyses of CRISPR/Cas9-mediated mutagenesis in mutant plants. **(A)** T7EI digestion assay of the mutants at the target sites. The PCR products for *Gh14-3-3d* from At, Dt, and total DNA (AtDt) were digested, response fragments in size (bp) were indicated on the right side. M, DNA molecular marker; T0–16 and T0–30 were represents plants for T7EI digestion assay. **(B)** The indels of *Gh14-3-3d* at target site of AtDt genome in T0–16 and T0–30 illustrated based on nucleotides and chromatograms by Sanger sequencing, respectively. The PAM sequence was shown in red. The PCR products were acquired by genome DNA and the universal primers, digested and sequenced for analyzing *Gh14-3-3d* gene editing.

FIGURE S6 A novel indel that occurred in T1. T0–6–8 with a new indel (d3) was an offspring plant segregated from T0–6 mutant.

TABLE S1 The PCR, RT-PCR, and qPCR primers.

TABLE S2 Mutations detected in the putative CRISPR/Cas9 off target sites in WT, *ce1*, and *ce2* plants.

DATA S1 The artificial synthesizing sequence of *sgRNA* expression cassettes in vector of pYLCRISPR/Cas9-CBD.
