# Supplementary figures and images for: Simultaneous Editing of Two Copies of Gh14-3-3d Confers Enhanced Transgene-Clean Plant Defense Against Verticillium dahliae in Allotetraploid Upland Cotton
Source: Front Plant Sci. 2018 Jun 28;9:842. doi: 10.3389/fpls.2018.00842 (PMC6036271; doi:10.3389/fpls.2018.00842)

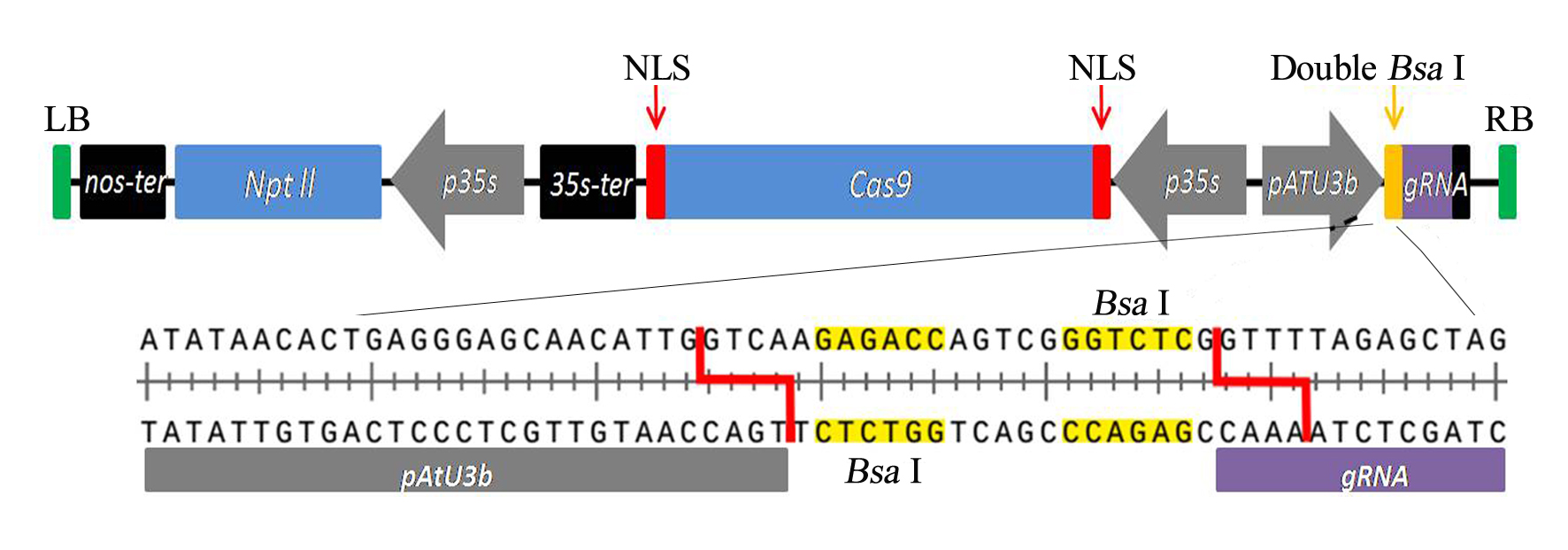

Supplement: FIGURE S1 — Schematic of the T-DNA region of plant gene editing vector pYLCRISPR/Cas9-CBD. The sequence of BsaI recognition motifs were highlighted in yellow, digestion site of BsaI were shown by red lines. [file Image_1.JPEG]

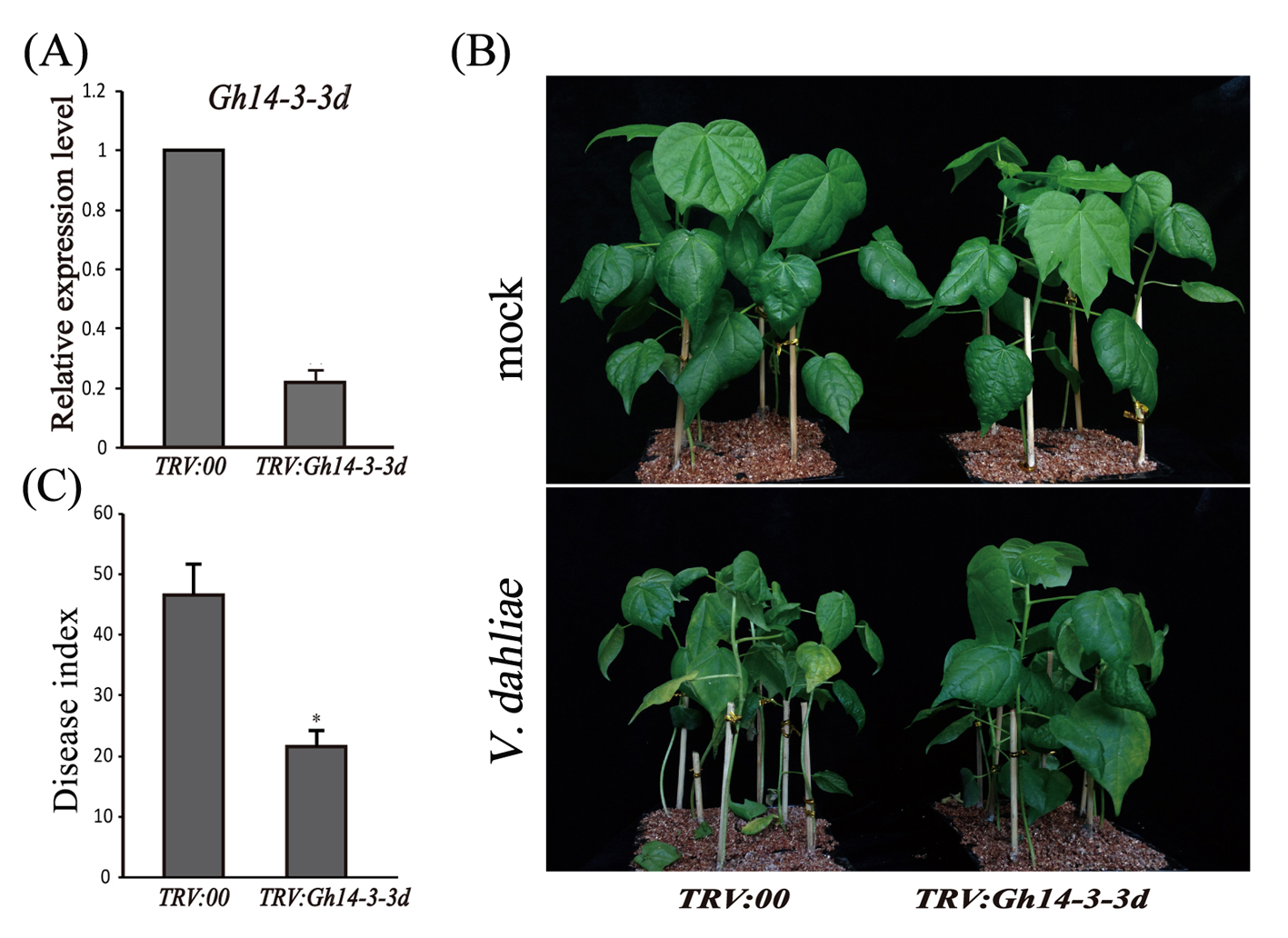

Supplement: FIGURE S2 — Increased resistance of the Gh14-3-3d-silenced plants to V. dahliae. (A) Gh14-3-3d expression levels in silenced plants (TRV: Gh14-3-3d) and the control (TRV: 00) were determined by qPCR. The Gh14-3-3d expression level of the control was designated 1, and the average expression level in Gh14-3-3d -silenced plants was determined from 15 independent tested plants (3 replicates and 5 plants per replicate). (B) Disease symptoms on Gb14-3-3d-silenced plants and the control at 10th day post-inoculated with V. dahliae. (C) The disease index of the Gh14-3-3d-silenced plants and the control. Error bars represent the SD of three biological replicates (n ≥ 36). Asterisk indicates statistically significant differences compared to the control using Student’s t-test (P < 0.05). [file Image_2.JPEG]

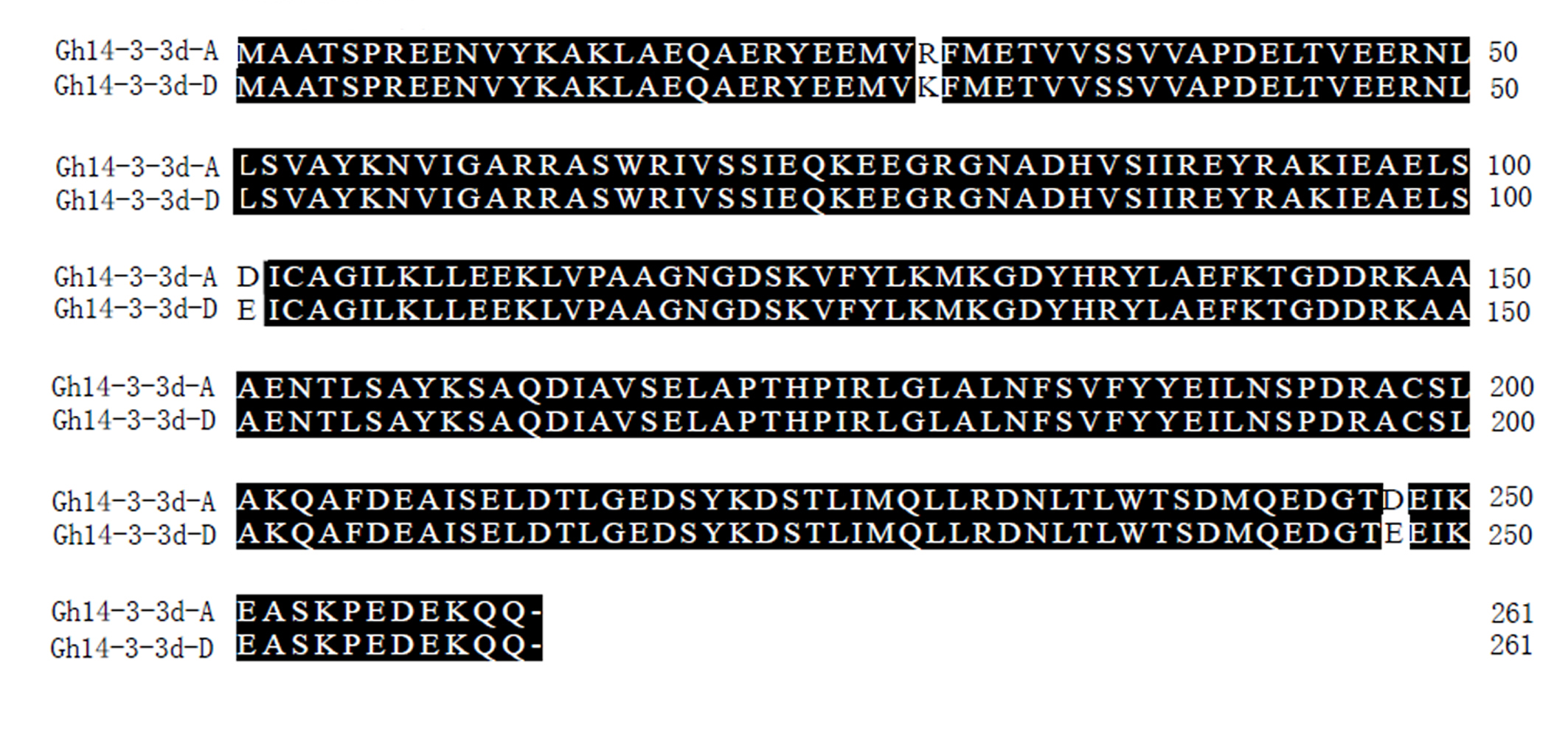

Supplement: FIGURE S3 — Amino acid alignment of the two copies of Gh14-3-3d encoding proteins, Gh14-3-3d-A and Gh14-3-3d-D. [file Image_3.JPEG]

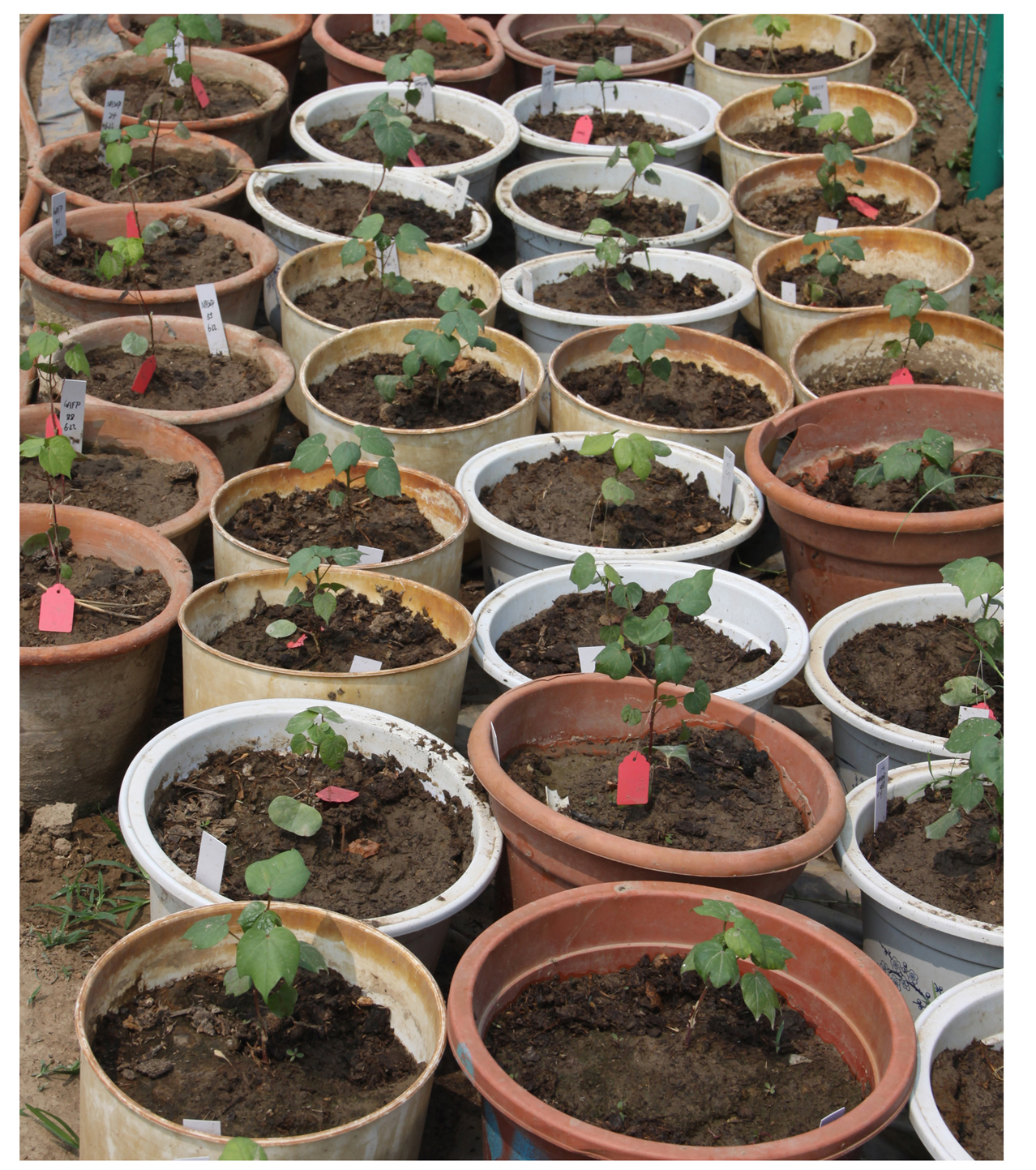

Supplement: FIGURE S4 — The putative transgenic plants in T0 in greenhouse. The regenerate plantlets were directly transplanted, or grafted on receptor seedlings in pots with 1:3 vermiculite and organic matter soil. [file Image_4.JPEG]

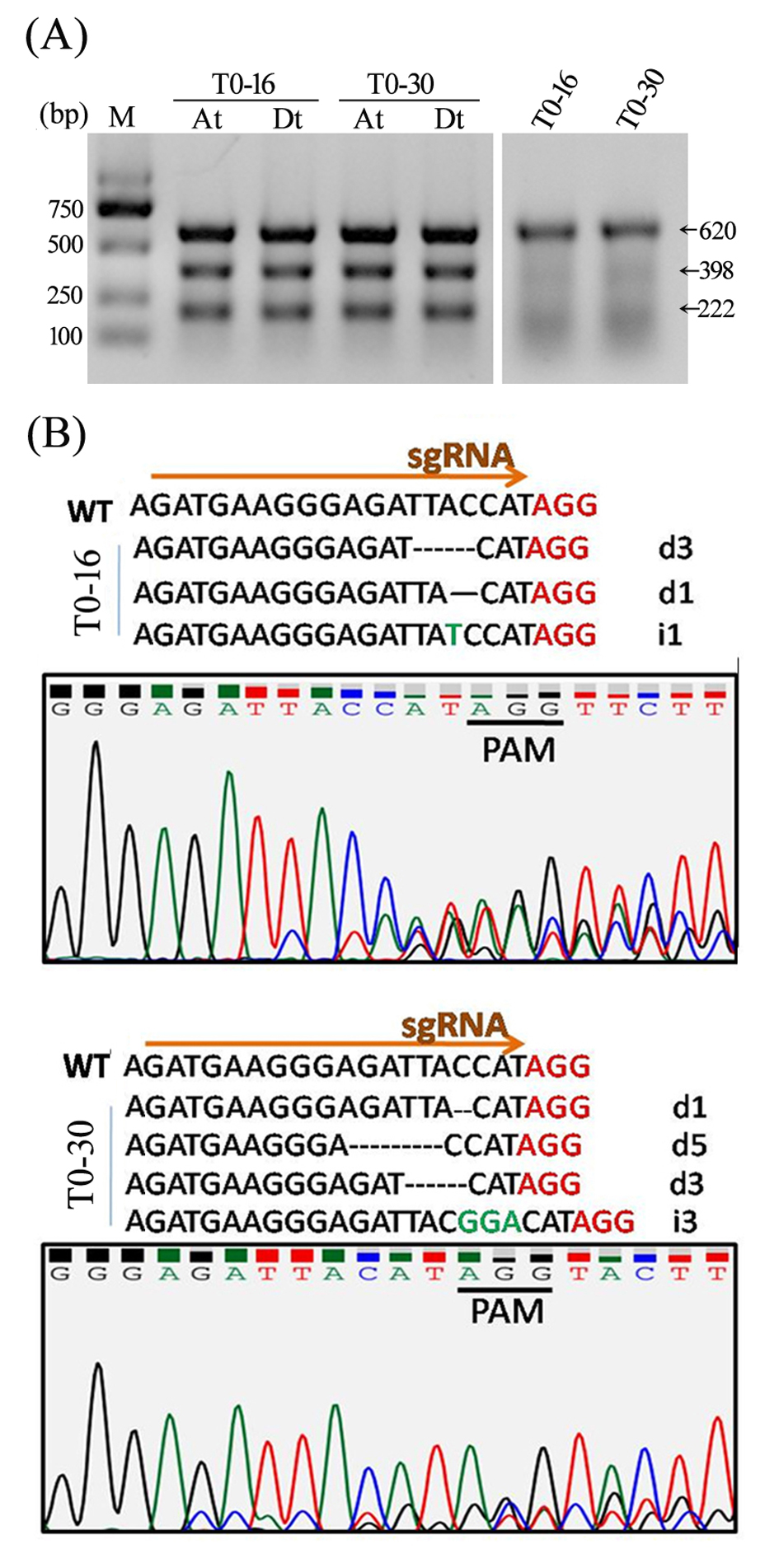

Supplement: FIGURE S5 — Two copies of Gh14-3-3d affect the analyses of CRISPR/Cas9-mediated mutagenesis in mutant plants. (A) T7EI digestion assay of the mutants at the target sites. The PCR products for Gh14-3-3d from At, Dt, and total DNA (AtDt) were digested, response fragments in size (bp) were indicated on the right side. M, DNA molecular marker; T0–16 and T0–30 were represents plants for T7EI digestion assay. (B) The indels of Gh14-3-3d at target site of AtDt genome in T0–16 and T0–30 illustrated based on nucleotides and chromatograms by Sanger sequencing, respectively. The PAM sequence was shown in red. The PCR products were acquired by genome DNA and the universal primers, digested and sequenced for analyzing Gh14-3-3d gene editing. [file Image_5.JPEG]

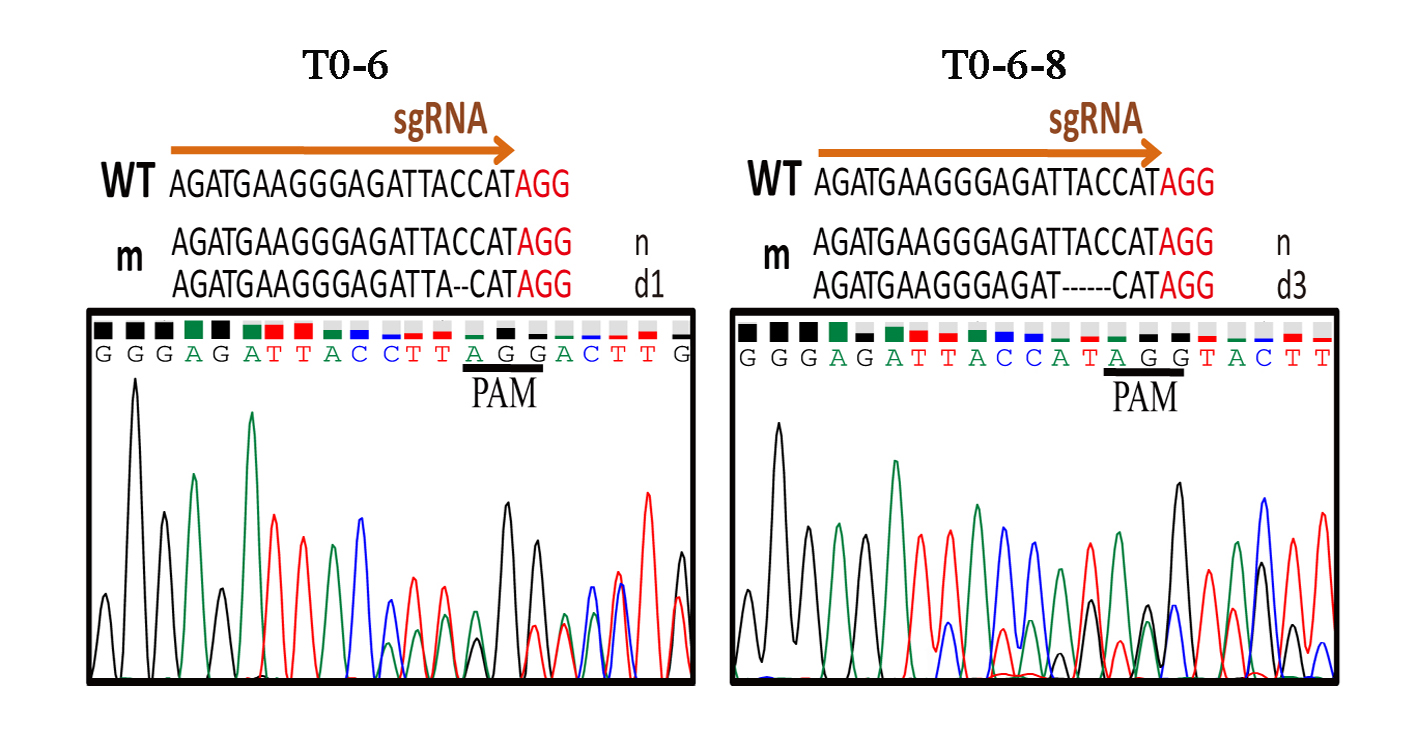

Supplement: FIGURE S6 — A novel indel that occurred in T1. T0–6–8 with a new indel (d3) was an offspring plant segregated from T0–6 mutant. [file Image_6.JPEG]
