# Supplementary material for: Simultaneous Editing of Two Copies of Gh14-3-3d Confers Enhanced Transgene-Clean Plant Defense Against Verticillium dahliae in Allotetraploid Upland Cotton
Source: Front Plant Sci. 2018 Jun 28;9:842. doi: 10.3389/fpls.2018.00842 (PMC6036271; doi:10.3389/fpls.2018.00842)
Supplement: TABLE S1 — The PCR, RT-PCR, and qPCR primers. [file Table_1.DOCX]

**Table S1.** The PCR, RT-PCR and qPCR primers

| primers | sequences （5’to 3’） | purpose |
| --- | --- | --- |
| *Gh14-3-3d-F* | GGGAGCAACATTGGTCAAGATGAAGGGAGATTACCAT | Target sequence inserted vector |
| *Gh14-3-3d-R* | CTATTTCTAGCTCTAAAACATGGTAATCTCCCTTCATCT |  |
| *CBD-F* | GGTAAGGCGCGCCGTAGTGCTCGATTTACTTTAAATTTTTTCTTATGC | sgRNA cassette inserted in vector |
| *CBD-R* | GAGGCGCGCCAATGATACCGTTGAAAAAAAAAGCACCGACTCGGT |  |
| *Gh14-3-3d-F1* | GCCGTGCTTCCTGGAGGATTG | VIGS vector construction |
| *Gh14-3-3d-R1* | GCGTATTCTCAGCAGCGGCTTTC |  |
| *PA* | TGGGAATAGGGTGTGGTTATCAAAGGATGCGGTTG | *Gh14-3-3dA/D* isolation |
| *PD* | TATTAAAATATCTTTATAAAAATTGTAGGTTCCACT |  |
| *PF* | CCGCTACTTCACCACGTGAGG |  |
| *PR* | CATAAACTTTCCATTGTAATTCAATGTTAT |  |
| *Gh14-3-3d-F2* | ACTTCTTCGTGATAACCTTACTTTGTGGAC | qPCR |
| *Gh14-3-3d-R2* | TCGAAGATTAGGGATTTTACTGTTGTT |  |
| *β-tubulin-F* | AACAACAGTCCGATGGATAATTC |  |
| *β-tubulin-R* | GTACCGGGCTCGAGATCG |  |
| *BZR1-F* | AGTCCAACCCATCGTCATTTCC |  |
| *BZR1-R* | CACAAGATTGAATGTTGGCGAAGT |  |
| *BRI1-F* | TGGAGACGACCCACAATGCTT |  |
| *BRI1-R* | CTGCTTACCTTCTGGGACTTCTTTT |  |
| *BIN2-F* | ACCTAACGCTCGCCTACCAAAT |  |
| *BIN2-R* | TGAAGTCCTATTTGCCGTTTCAC |  |
| *PDF1.2-F* | CTGTGGTAGCGGATGGTGATAAG |  |
| *PDF1.2-R* | GTGCAGACGCATTTGCGAAGGAA |  |
| *PR4-F* | GAGGGTAAGAAACTCAAGGACTGG |  |
| *PR4-R* | CTCCATCAGTGTCCAATCGGTT |  |
| *UB7-F* | GAAGGCATTCCACCTGACCAAC |  |
| *UB7-R* | CTTGACCTTCTTCTTCTTGTGCTTG |  |
| *Cas9-F* | ATCACCACCAAGCTGGGAAAG | RT-PCR |
| *Cas9-R* | AAGGTCCTTTCTATGCCTCAGGTT |  |
| *Gh_A13G0606-F* | CATGGAGACCGTCGTCTCCG | PCR |
| *Gh_A13G0606-R* | CCCTCCGGTGTCAGGTAGCC |  |
| *Gh_D04G1522-F* | CGAACTCTCCGTCGAGGAAC |  |
| *Gh_D04G1522-R* | ACACAATGGAAAGCAGATCCG |  |
| *Gh_D13G0518-F* | TCTTCCAATCCAGACAGCACC |  |
| *Gh_D13G0518-R* | TGCCTTCCGCTTATGTTTCC |  |
| *Gh_A04G0978-F* | CCGTGACTACAGGGCCAAGA |  |
| *Gh_A04G0978-R* | TGGCCAATAATCCAAAGCAAC |  |
| *Gh_A05G2280-F* | GACTCGCGAGGAACACGTCT |  |
| *Gh_A05G2280-R* | CCGACACGTCAATCGACTGT |  |
| *Gh_D05G2540-F* | CGAGCGCTACGACGAAATG |  |
| *Gh_D05G2540-R* | ATGCCTGTCCCGATCCAAG |  |
| *Gh_D05G2122-F* | CGGAAGCTCACCAATTTCTGC |  |
| *Gh_D05G2122-R* | CACCGAGTAATTCAGCGCAAG |  |
| *Gh_A05G1888-F* | CGGAAGCTCACCAATTTCTGC |  |
| *Gh_A05G1888-R* | ATCGGGTGCGTTGGTGCTA |  |
| *NPTII-F* | CGTAAAGCACGAGGAAGCGGTCAG |  |
| *NPTII-R* | ACTGGGCACAACAGACAATCGGCTG |  |
| *Fr1-F* | GGACGTTTTTAATGTACTGAATTAACGCC |  |
| *Fr1-R* | TCCGGTGCCCTGAATGAACTCC |  |
| *Fr2-F* | TTGGAGAGCTGAAGCTTAGCGTCC |  |
| *Fr2-R* | CATTTCATTTGGAGAGGACGTCGAGAG |  |
| *Fr3-F* | AAGGCGCGCCGTAGTGCTCGATT |  |
| *Fr3-R* | GTGCTGCAAGGCGATTAAGTTGGG |  |
