# Supplementary material for: Simultaneous Editing of Two Copies of Gh14-3-3d Confers Enhanced Transgene-Clean Plant Defense Against Verticillium dahliae in Allotetraploid Upland Cotton
Source: Front Plant Sci. 2018 Jun 28;9:842. doi: 10.3389/fpls.2018.00842 (PMC6036271; doi:10.3389/fpls.2018.00842)
Supplement: TABLE S2 — Mutations detected in the putative CRISPR/Cas9 off target sites in WT, ce1, and ce2 plants. [file Table_2.DOCX]

**Table S2 Mutations detected in the putative CRISPR/Cas9 off target sites in WT, ce1 and ce2 plants**

| Putative  off-target locus | Gene accession number | No. of  mismatching bases | Real sequence of  off-target site in WT, *ce1* and *ce2* | Difference of sequence * |  | |
| --- | --- | --- | --- | --- | --- | --- |
| A13:+14552450 | Gh_A13G0606 | 1 | AAATGAAGGGAGATTACCATAGG | No |  |  |
| D04:+47252849 | Gh_D04G1522 | 1 | AAATGAAGGGAGATTACCATAGG | No |  |  |
| D13:-6707194 | Gh_D13G0518 | 2 | AAATGAAGGGAGATTACCATAGG | No |  |  |
| A04:+59431360 | Gh_A04G0978 | 2 | AAATGAAAGGAGATTACCATAGG | No |  |  |
| A05:-26848945 | Gh_A05G2280 | 1 | AGATGAAGGGAGATTATCATAGG | No |  |  |
| D05:-25686633 | Gh_D05G2540 | 2 | AGATGAAGGGCGATTATCATAGG | No |  |  |
| D05:+19857533 | Gh_D05G2122 | 3 | AGATGAAAGGGGATTATCATCGG | No |  |  |
| A05:+19797827 | Gh_A05G1888 | 3 | AGATGAAAGGGGATTATCATCGG | No |  |  |
| A13:+12424236  scaffold49004:-64  D04:+12843222  D02:+16970265  A13:-68291928  A07:-50561443  D05:-12296149  A11:+85314493  D11:+20642183 | ISe  ISe  ISe  ISe  ISe  ISe  ISe  ISe  ISe | 2  2  2  3  4  4  4  4  4 | AAATGAAGAGAGATTACCATAGG  AAATGAAGAGAGATTACCATAGG  AAATGAAGGGAGATTACCAAAGG  TGATGTAGGGACATTACCATCGG  GTATGAAGGGAAAATACCATCGG  AGATGAAAGAAGGTTACCAAAGG  AGAAAGAGGAAGATTACCATAGG  AGATAAAAAGAGATTAACATAGG  TGATGTAGAGACATTACCATTGG | NT  NT  NT  NT  NT  NT  NT  NT  NT |  |  |

Note: *, Difference of putative and real off-target sequence; putative off-target sequence comes from the cotton gene database, real off-target sequence came from sequencing of PCR products in WT, *ce1* and *ce2*. Nucleotides in red were mismatched with the on-target sequence. NO, there was no difference between putative and real off-target sequence. NT, no test.
