# Supplementary material for: Simultaneous Editing of Two Copies of Gh14-3-3d Confers Enhanced Transgene-Clean Plant Defense Against Verticillium dahliae in Allotetraploid Upland Cotton
Source: Front Plant Sci. 2018 Jun 28;9:842. doi: 10.3389/fpls.2018.00842 (PMC6036271; doi:10.3389/fpls.2018.00842)
Supplement: DATA S1 — The artificial synthesizing sequence of sgRNA expression cassettes in vector of pYLCRISPR/Cas9-CBD. [file Data_Sheet_1.DOCX]

**Data S1 The artificial synthesizing sequence of *sgRNA* expression cassettes in vector of pYLCRISPR/Cas9-CBD**

ggtaaggcgcgccgtagtgCTCGAtttactttaaattttttcttatgcagcctgtgatggataactgaatcaaacaaatggcgtctgggtttaagaagatctgttttggctatgttggacgaaacaagtgaacttttaggatcaacttcagtttatatatggagcttatatcgagcaataagataagtgggctttttatgtaatttaatgggctatcgtccatagattcactaatacccatgcccagtacccatgtatgcgtttcatataagctcctaatttctcccacatcgctcaaatctaaacaaatcttgttgtatatataacactgagggagcaacattggtcaagagaccAGTCGggtctcggttttagagctagaaatagcaagttaaaataaggctagtccgttatcaacttgaaaaagtggcaccgagtcggtgctttttttTTcaacggtatcattggcgcgcctc

> *sgRNA* expression cassettes in pYLCRISPR/Cas9-CBD

ggtaaggcgcgccgtagtgCTCGAtttactttaaattttttcttatgcagcctgtgatggataactgaatcaaacaaatggcgtctgggtttaagaagatctgttttggctatgttggacgaaacaagtgaacttttaggatcaacttcagtttatatatggagcttatatcgagcaataagataagtgggctttttatgtaatttaatgggctatcgtccatagattcactaatacccatgcccagtacccatgtatgcgtttcatataagctcctaatttctcccacatcgctcaaatctaaacaaatcttgttgtatatataacactgagggagcaacattggtcaagagaccAGTCGggtctcggttttagagctagaaatagcaagttaaaataaggctagtccgttatcaacttgaaaaagtggcaccgagtcggtgctttttttTTcaacggtatcattggcgcgcctc

The sequence of AtU3b promotor, *sgRNA* and two *Bsa*I recognition motifs were highlighted in green, yellow and red, respectively.
